# Investigation of progression pattern and associated risk factors in glaucoma patients with initial paracentral scotomas using Humphrey 10-2

**DOI:** 10.1038/s41598-021-97446-6

**Published:** 2021-09-20

**Authors:** Eun Kyoung Kim, Hae-Young Lopilly Park, Kyung Euy Hong, Da Young Shin, Chan Kee Park

**Affiliations:** 1 Department of Ophthalmology, Seoul St. Mary’s Hospital, 505 Banpo-dong, Seocho-ku, Seoul, 137-701 Korea; 2 College of Medicine, The Catholic University of Korea, Seoul, Republic of Korea

**Keywords:** Eye diseases, Risk factors

## Abstract

Central visual field (VF) progression could directly threaten patientss visual function compared to glaucomatous damage. This study was designed to investigate visual field (VF) progression pattern and associated risk factors including optical coherence topography angiographic (OCT-A) findings in glaucoma patients with initial paracentral scotoma. This prospective, observational study included 122 eyes presenting as initial paracentral scotomas with serial 24-2 and 10-2 VF tests at the glaucoma clinic of Seoul St Mary's Hospital between November 2017 and August 2020. The participants underwent at least 5 serial VF exams and OCT-A at baseline. Numerical values of the initial and final 10-2 VF tests were averaged for each VF test point using the total deviation map. Innermost 10-2 VF progression was defined as three or more new contiguous points at the central 12 points on 10-2 VF. Other clinical characteristics were collected including history of disc hemorrhage and vessel density (VD) was measured from OCT-A images. Linear regression analysis was performed to obtain the change of mean deviation and a cut-off for progression was defined for both 24-2 and 10-2 VFs. The average total deviation maps of the initial 10-2 VF tests shows initial paracentral scotoma located in the superior region in an arcuate pattern that was deep in the 4°–6° region above fixation. This arcuate pattern was more broadly located in the 4°–10° region in the primary open-angle glaucoma (POAG) group, while it was closer to fixation in 0°–4° region in the normal-tension glaucoma (NTG) group. The final average map shows deepening of scotomas in the 4°–10° region in POAG, which deepened closer to the region of fixation in NTG. The diagnosis of NTG (β 1.892; 95% CI 1.225–2.516; *P* = 0.035) and lower choroidal VD in the peripapillary atrophy (PPA) region (β 0.985; 95% CI 0.975 to 0.995; *P* = 0.022) were significantly related to innermost 10-2 VF progression. Initial paracentral scotomas in NTG tended to progress closer to the region of fixation, which should be monitored closely. Important progression risk factors related to paracentral scotoma near the fixation were the diagnosis of NTG and reduced choroidal VD in the β-zone PPA region using OCT-A. We should consider vascular risk factors in NTG patients presenting with initial paracentral scotoma to avoid vision threatening progression of glaucoma.

## Introduction

Glaucomatous visual field (VF) damage usually occurs the 10°–30° region of the VF^1^. However, recent studies have reported that paracentral damage within the 10° region of the VF, including macular involvement, occur in early stage of glaucoma^2,3^. The VF tests commonly used to assess glaucomatous functional loss, the 24-2 or 30-2 threshold test of standard automated perimetry (SAP), have been reported to underestimate damage in the central 10° region^4–6^. Furthermore, more than 30% of the retinal ganglion cells exist within the central 10° region of the VF, and only four points from SAP 24-2 or SAP 30-2 fall within this region. Several studies have suggested the importance of using central SAP 10-2, with 68 points spaced 2° apart in the same region^7–10^. Glaucoma patients with vascular risk factors show VF defects in the central 10° region even at the early stage of the disease^11–13^. Central VF progression was reported to be related to vascular risk factors, including disc hemorrhage, autonomic dysfunction, migraine, orthostatic hypotension, and Raynaud’s phenomenon^14,15^. Using optical coherence tomography (OCT) angiography, central VF involvement has been reported to be associated with vessel density (VD) loss^16–19^. Central VF progression could directly threaten patients’ visual function compared to glaucomatous damage, which usually involves the 10°–30° region. Therefore, it is important to investigate the pattern of VF progression within the central 10° region and the role of vascular risk factors and findings from OCT angiography in central VF progression.

We prospectively performed SAP 24-2 and 10-2 tests in glaucoma patients with initial paracentral scotoma. Analysis of the progression pattern using SAP 10-2 VF was performed and risk factors, including OCT angiographic findings associated with central VF progression, were investigated.

## Results

**Figure 1:**
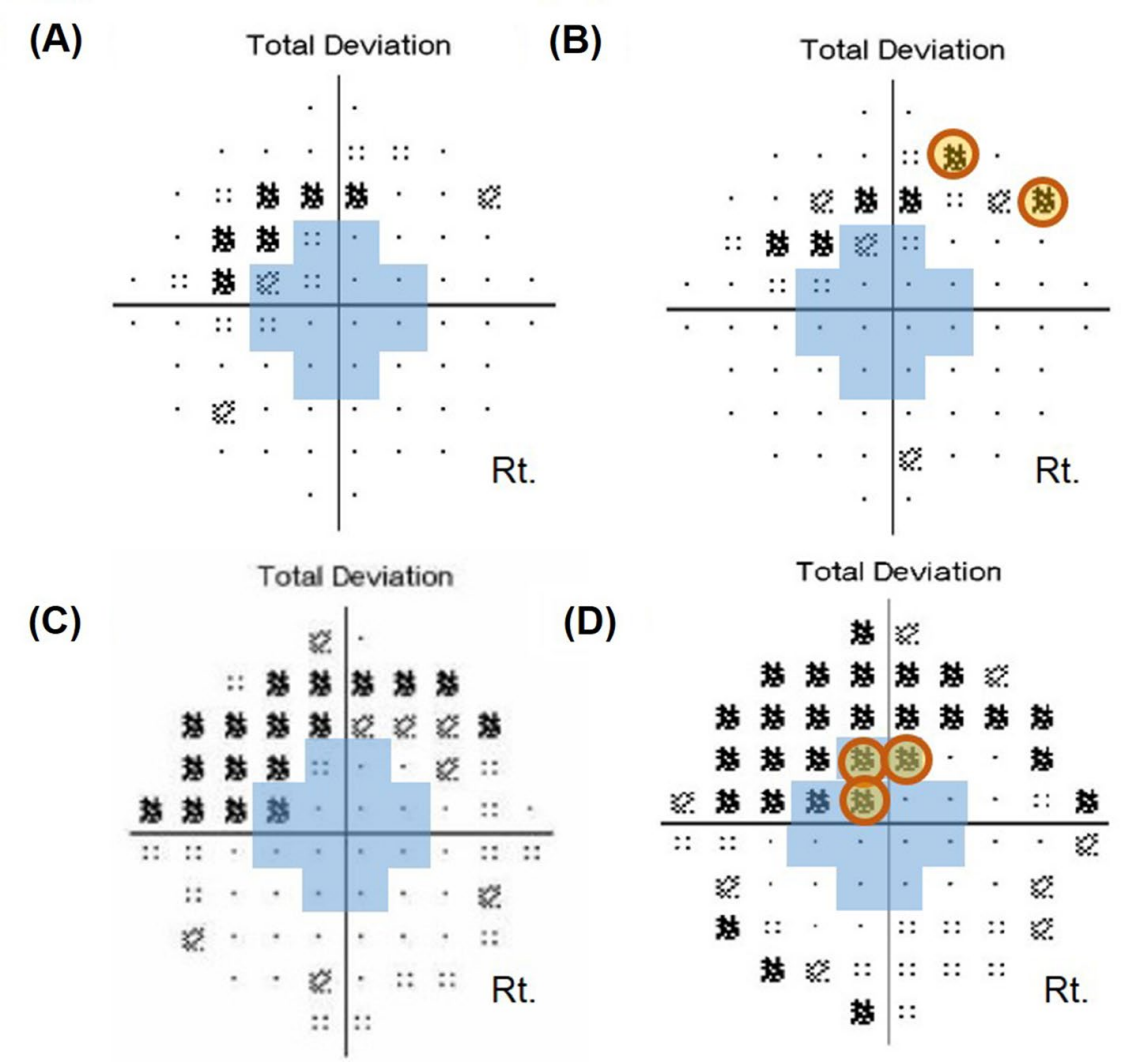
Classification of progression of initial paracentral scotomas according to location on the 10-2 visual field (VF) test. (**A**) and (**B**) Compared to outermost 10-2 VF progression, (**C**) and (**D**) three or more new contiguous points (5%, 5%, and 1% or 5%, 2%, and 2% depressed from normative database) in the central 12 points on 10-2 VF were considered as innermost 10-2 VF progression.

A total of 156 glaucoma patients underwent both serial 24-2 and 10-2 VF tests. Among these patients, a total of 122 eyes of 122 glaucoma patients had reliable VF tests and OCT-A images to be analyzed. Total of mean follow-up period was 2.32 ± 0.16 years. Among included patients, 71 eyes were diagnosed as NTG and 51 eyes were diagnosed as POAG. Central corneal thickness was significantly thinner in the NTG eyes than the POAG eyes (*P* = 0.010). Other demographic characteristics were similar between the two groups (Table 1). The cases were classified as progression of initial paracentral scotomas are shown in Fig. 1. The intraclass correlation coefficient (ICC) ranges of the interobserver evaluations were 0.89–0.95 for vessel density (VD) calculation from OCT-A images (Fig. 1).

**Table 1:** Baseline characteristics of glaucoma patients who underwent serial 24-2 and 10-2 visual field tests.

	Total	POAG	NTG	*P* value
**Demographics characteristics**				
Age (years)	54.59 ± 12.79	50.41 ± 16.93	55.71 ± 11.56	0.126^a^
Sex (female:male)	77:45	33:18	44:27	0.454^b^
Mean follow-up period (years)	2.32 ± 0.16	2.25 ± 0.19	2.41 ± 0.17	0.817^a^
Used eye drops (n)	1.14 ± 0.35	1.20 ± 0.40	1.19 ± 0.40	0.988^a^
**Ocular characteristics**				
Spherical equivalent (diopters)	− 2.71 ± 3.25	− 3.02 ± 2.67	− 2.62 ± 3.42	0.709^a^
Axial length (mm)	24.89 ± 1.37	25.24 ± 1.23	24.79 ± 1.42	0.323^a^
Untreated baseline IOP (mmHg)	19.12 ± 2.33	23.27 ± 2.54	17.89 ± 2.01	0.043^a^
Mean treated IOP during follow-up (mmHg)	16.22 ± 2.98	17.79 ± 1.76	15.16 ± 2.94	0.157^a^
Central corneal thickness (μm)	528.34 ± 31.72	549.90 ± 22.96	523.38 ± 31.54	0.010^a^
Presence of disc hemorrhage, n (%)	30 (24.6)	14 (27.5)	16 (22.5)	0.263^b^
**OCT parameters**				
Average RNFL thickness (μm)	78.42 ± 10.55	79.70 ± 11.13	78.00 ± 10.50	0.554^a^
Average GC/IPL thickness (μm)	69.67 ± 7.83	72.00 ± 70.72	69.03 ± 7.02	0.337^a^
Minimum GC/IPL thickness (μm)	53.76 ± 9.83	54.00 ± 17.58	53.66 ± 7.50	0.908^a^
**VF parameters**				
Performed SAP 24-2 (n)	5.65 ± 1.03	6.03 ± 1.34	5.97 ± 1.15	0.118^a^
MD of SAP 24-2 (dB)	− 3.31 ± 2.46–3.31 ± 2.46	− 3.70 ± 2.21	− 3.22 ± 2.54	0.472^a^
MD slope of SAP 24-2 (dB/years)	0.29 ± 1.04	− 0.11 ± 1.85	0.28 ± 1.03	0.230^a^
PSD of SAP 24-2 (dB)	5.11 ± 3.17	5.74 ± 3.48	4.81 ± 3.07	0.109^a^
Performed SAP 10-2 (n)	5.06 ± 0.24	5.08 ± 0.28	5.06 ± 0.245.06 ± 0.24	0.596^a^
MD of SAP 10-2 (dB)	− 5.48 ± 3.25	− 5.86 ± 2.97	− 5.36 ± 3.34	0.575^a^
MD slope of SAP 10-2 (dB/years)	− 0.27 ± 1.23	− 0.11 ± 1.85	− 0.33 ± 0.95	0.107^a^
PSD of SAP 10-2 (dB)	7.32 ± 3.97	8.19 ± 3.65	7.09 ± 4.06	0.312^a^
**OCT angiography parameters**				
Optic disc, superficial VD (%)	36.94 ± 8.86	34.42 ± 7.75	36.94 ± 8.79	0.438^a^
Parapapillary atrophy, choroidal VD (%)	51.73 ± 20.23	60.53 ± 17.82	49.66 ± 19.88	0.140^a^
Macular superficial VD (%)	33.51 ± 3.86	32.45 ± 2.03	33.62 ± 3.89	0.353^a^
Macular deep VD (%)	47.03 ± 6.91	45.64 ± 1.14	47.08 ± 7.49	0.549^a^

IOP, intraocular pressure; RNFL, retinal nerve fiber layer; mGC/IPL, macular ganglion cell/inner plexiform layer; SAP, standard automated perimetry; MD, mean deviation; PSD, pattern standard deviation; dB, decibels; POAG, primary open-angle glaucoma; NTG, normal-tension glaucoma.

^a^Student *t*-test.

^b^*Chi-square* test.

**Figure 2:**
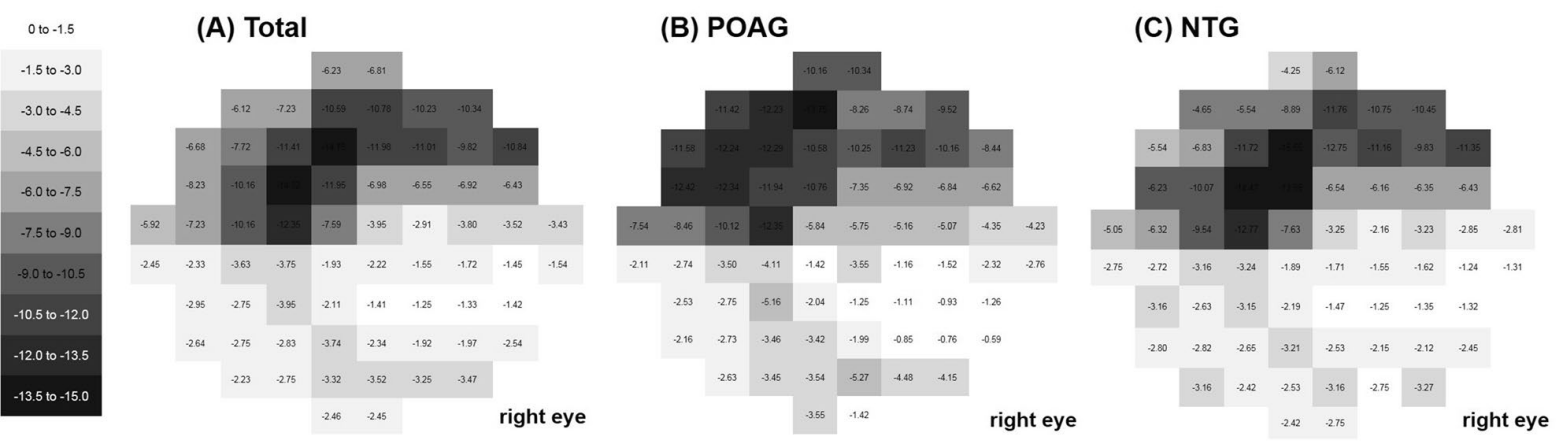
Cross-sectional analysis of the total 10-2 visual tests (VF) during the whole follow-up period. For cross-sectional analysis to show the location and pattern of the paracentral scotoma, numerical values of the total 10-2 VF tests during the whole follow-up period were averaged for each VF test point using the total deviation map, generating one average map for each subgroup. A linear grayscale was generated and applied to all maps. (**A**) Scotoma was located in the superior region in an arcuate pattern that was deep in the 4°–6° region above the region of fixation. (**B**) In the primary open-angle glaucoma group, this arcuate pattern was more broadly located in the 4°–10° region above the region of fixation. (**C**) However, the scotomas were more closely located around the region of fixation in the 0°–4° region in the normal-tension glaucoma group.

**Figure 3:**
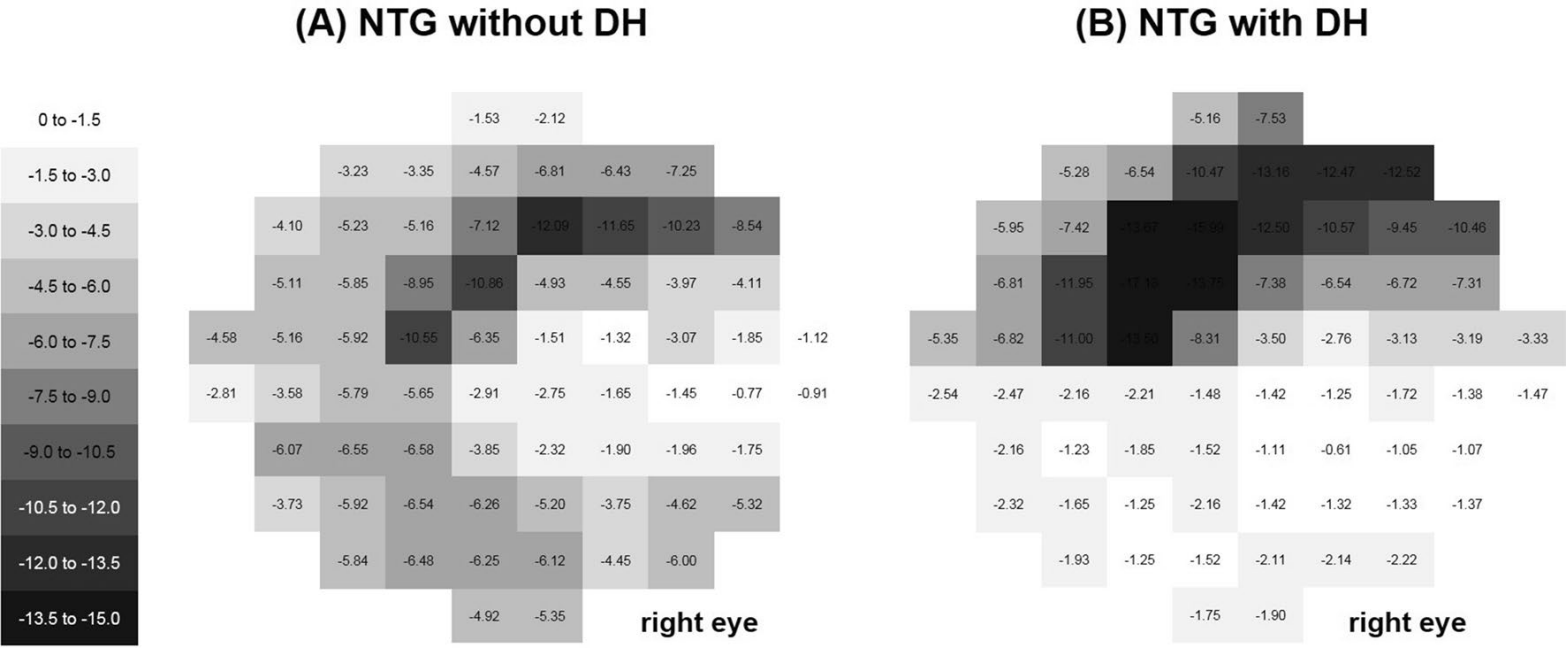
Cross-sectional analysis of the initial paracentral scotoma on 10-2 visual tests (VF) according to the presence or absence of disc hemorrhage. Numerical values of the first 10-2 VF test on the total deviation map were averaged for each VF test location, generating one average map for each subgroup at initial presentation. A linear grayscale was generated and applied to all maps. A and B. Normal-tension glaucoma with disc hemorrhage had initial paracentral scotomas deeper and greater near the region of fixation compared to those without disc hemorrhage.

**Figure 4:**
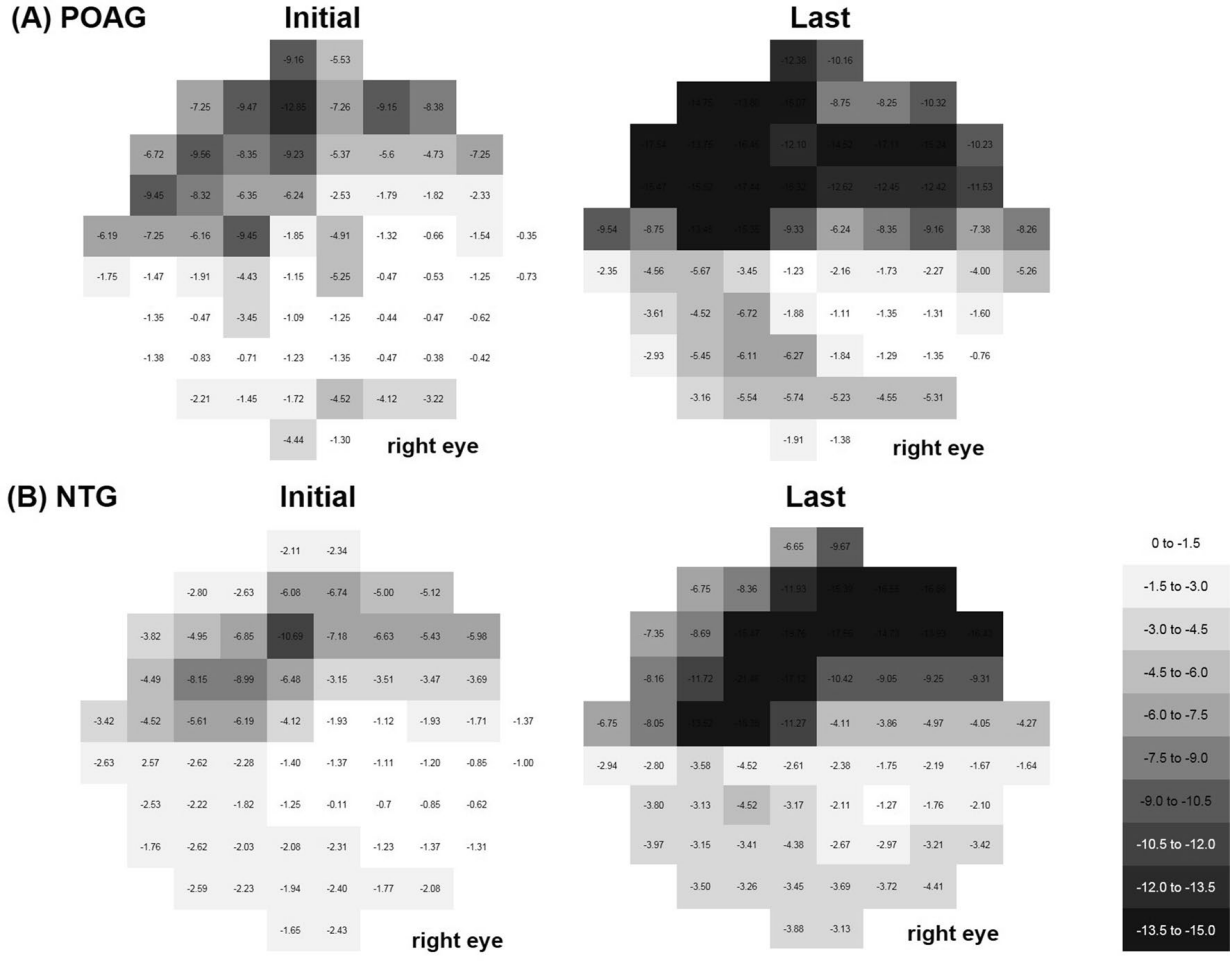
Longitudinal analysis of the initial paracentral scotoma to examine the pattern of progression. The average total deviation maps of the initial and final 10-2 VF tests for each subgroup are shown. A and B. The progression pattern of the initial paracentral scotoma of the primary open-angle glaucoma was deepened in the 4°–10° region, which deepened closer to the region of fixation in normal-tension glaucoma in the 0°–4° region.

The average total deviation maps of the total 10-2 VF tests for each subgroup are shown in Fig. 2. First, we generated an average map using all of the reliable 10-2 VF tests. The paracentral scotoma was located in the superior region in an arcuate pattern that was deep in the 4°–6° region above fixation (Fig. 2A). This arcuate pattern was more broadly located in the 4°–10° region above the region of fixation in the POAG group (Fig. 2B), while the scotomas were closer to the region of fixation in the 0°–4° region in the NTG group (Fig. 2C). We generated average total deviation maps in NTG eyes according to the presence or absence of disc hemorrhage, and the results indicated that the paracentral scotomas were larger and deeper in the 0°–6° region close to the fixation in NTG with disc hemorrhage (Fig. 3B) than without disc hemorrhage (Fig. 3A). Generation of average total deviation maps using 10-2 VFs at the initial and final visits could show the progression pattern of the initial paracentral scotomas. The average map shows deepening of scotomas in the 4°–10° region in POAG (Fig. 4A), which deepened closer to the region of fixation in NTG (Fig. 4B).

Among 122 eyes, 17 (13.9%) showed VF progression on 24-2 VF test. Risk factors related to 24-2 VF progression are shown in Table 2. The diagnosis of NTG (β, 3.540; 95% confidence interval (CI), 1.752 to 5.320; *P* = 0.016), lower choroidal VD in the peripapillary atrophy (PPA) region (β, 0.873; 95% CI, 0.794 to 0.934; *P* = 0.042), and lower macular superficial VD (β, 0.702; 95% CI, 0.527 to 0.877; *P* = 0.011) were significantly related to 24-2 VF progression. Risk factors related to 10-2 VF progression are shown in Table 3. Among 122 eyes, 26 (21.3%) showed VF progression on 10-2 VF test. Presence of disc hemorrhage (β, 5.259; 95% CI, 1.816 to 9.245; *P* = 0.033), thinner macular GC/IPL thickness (β, 0.724; 95% CI, 0.545–0.926; *P* = 0.032), and lower choroidal VD in the PPA region (β, 0.816; 95% CI, 0.795 to 0.890; *P* = 0.016) were significantly related to 10-2 VF progression. Among 122 eyes, 24 (19.7%) showed VF progression in the innermost region on 10-2 VF test. There was significant difference between the progression rate in the innermost region between NTG (17 eyes; 23.9%) and POAG (7 eyes; 13.7%, *P* = 0.031). The diagnosis of NTG (β, 1.892; 95% CI, 1.225 to 2.516; *P* = 0.035) and lower choroidal VD in the PPA region (β, 0.985; 95% CI, 0.975 to 0.995; *P* = 0.022) were significantly related to innermost 10-2 VF progression (Table 4).

**Table 2:** Associated risk factors to 24-2 VF progression in glaucoma patients.

Variables	Univariate		Multivariate	
	β (95% CI)	*P* value	β (95% CI)	*P* value
Age (years)	0.983 (0.947–1.029)	0.892		
Gender (female)	1.026 (0.892–1.117)	0.630		
Diagnosis of NTG	2.765 (1.033–4.126)	0.005	3.540 (1.752–5.320)	0.016
Axial length (mm)	0.803 (0.485–1.312)	0.344		
Untreated baseline IOP (mmHg)	1.027 (0.983–1.532)	0.615		
Mean treated IOP during follow-up (mmHg)	1.011 (0.997–1.023)	0.572		
Central corneal thickness (μm)	0.990 (0.972–1.007)	0.212		
Presence of disc hemorrhage, n (%)	1.923 (0.746–3.125)	0.112	1.992 (0.760–3.397)	0.203
Average RNFL thickness (μm)	0.985 (0.947–1.045)	0.595		
Average GC/IPL thickness (μm)	0.932 (0.874–0.995)	0.126	1.021 (0.981–1.046)	0.182
MD of SAP 24-2 (dB)	0.912 (0.850–1.044)	0.135	0.723 (0.450–1.045)	0.326
PSD of SAP 24-2 (dB)	0.963 (0.865–1.095)	0.525		
Optic disc, superficial VD (%)	0.982 (0.875–1.126)	0.313		
Parapapillary atrophy, choroidal VD (%)	0.943 (0.862–1.022)	0.053	0.873 (0.794–0.934)	0.042
Macular superficial VD (%)	0.815 (0.677–0.983)	0.026	0.702 (0.527–0.877)	0.011
Macular deep VD (%)	0.985 (0.924–1.045)	0.630		
Follow-up period (years)	1.023 (0.942–1.100)	0.547		

Variables with *P* < 0.2 were entered in the multivariate analysis.

NTG, normal-tension glaucoma; IOP, intraocular pressure; RNFL, retinal nerve fiber layer; GC/IPL, ganglion cell/inner plexiform layer; MD, mean deviation; PSD, pattern standard deviation; VD, vessel density.

**Table 3:** Associated risk factors to 10-2 VF progression in glaucoma patients.

Variables	Univariate		Multivariate	
	β (95% CI)	*P* value	β (95% CI)	*P* value
Age (years)	1.052 (1.033–1.089)	0.022	1.145 (0.916–1.272)	0.311
Gender (female)	1.716 (0.729–1.328)	0.256		
Diagnosis of NTG	3.312 (1.111–4.101)	0.047	5.323 (2.356–9.148)	0.272
Axial length (mm)	1.450 (1.298–1.622)	0.023	2.626 (0.950–5.114)	0.373
Untreated baseline IOP (mmHg)	1.005 (0.998–1.008)	0.325		
Mean treated IOP during follow-up (mmHg)	1.018 (0.991–1.326)	0.622		
Central corneal thickness (μm)	1.005 (0.992–1.019)	0.616		
Presence of disc hemorrhage, n (%)	3.645 (0.816–7.812)	0.047	5.259 (1.816–9.245)	0.033
Average RNFL thickness (μm)	1.031 (0.991–1.073)	0.149	1.163 (0.934–1.450)	0.254
Average GC/IPL thickness (μm)	1.045 (0.990–1.023)	0.105	0.724 (0.545–0.926)	0.032
MD of SAP 10-2 (dB)	1.045 (0.923–1.189)	0.512		
PSD of SAP 10-2 (dB)	1.059 (0.945–1.078)	0.259		
Optic disc, superficial VD	1.011 (0.961–1.068)	0.645		
Parapapillary atrophy, choroidal VD	0.835 (0.715–0.947)	0.045	0.816 (0.795–0.890)	0.016
Macular superficial VD	0.985 (0.858–1.134)	0.847		
Macular deep VD	0.945 (0.888–1.042)	0.454		
Follow-up period (years)	1.016 (0.955–1.120)	0.560		

NTG, normal-tension glaucoma; IOP, intraocular pressure; RNFL, retinal nerve fiber layer; GC/IPL, ganglion cell/inner plexiform layer; MD, mean deviation; PSD, pattern standard deviation; VD, vessel density.

Variables with *P* < 0.2 were entered in the multivariate analysis.

**Table 4:** Associated risk factors to innermost 10-2 VF progression in glaucoma patients.

Variables	Univariate		Multivariate	
	β (95% CI)	*P* value	β (95% CI)	*P* value
Age (years)	1.054 (1.016–1.054)	0.112	1.065 (0.960–1.179)	0.255
Gender (female)	1.074 (0.385–2.990)	0.912		
Diagnosis of NTG	1.735 (1.111–2.245)	0.030	1.892 (1.225–2.516)	0.035
Axial length (mm)	1.478 (0.220–3.016)	0.057	2.765 (1.112–4.255)	0.216
Untreated baseline IOP (mmHg)	1.021 (0.992–1.118)	0.581		
Mean treated IOP during follow-up (mmHg)	1.003 (0.995–1.008)	0.710		
Central corneal thickness (μm)	1.007 (0.994–1.025)	0.520		
Presence of disc hemorrhage, n (%)	2.322 (0.992–3.966)	0.075	2.410 (0.926–3.247)	0.072
Average RNFL thickness (μm)	1.025 (0.973–1.078)	0.350		
Average GC/IPL thickness (μm)	1.083 (0.988–1.180)	0.097	0.941 (0.856–1.012)	0.546
MD of SAP 10-2 (dB)	1.095 (0.943–1.269)	0.455		
PSD of SAP 10-2 (dB)	1.005 (0.890–1.131)	0.890		
Optic disc, superficial VD	1.052 (0.970–1.116)	0.245		
Parapapillary atrophy, choroidal VD	1.025 (0.992–1.055)	0.123	0.985 (0.975–0.995)	0.022
Macular superficial VD	0.910 (0.782–1.122)	0.511		
Macular deep VD	0.948 (0.865–1.040)	0.472		
Follow-up period (years)	1.022 (0.998–1.037)	0.730		

Variables with *P* < 0.2 were entered in the multivariate analysis.

NTG, normal-tension glaucoma; IOP, intraocular pressure; RNFL, retinal nerve fiber layer; GC/IPL, ganglion cell/inner plexiform layer; MD, mean deviation; PSD, pattern standard deviation; VD, vessel density.

**Figure 5:**
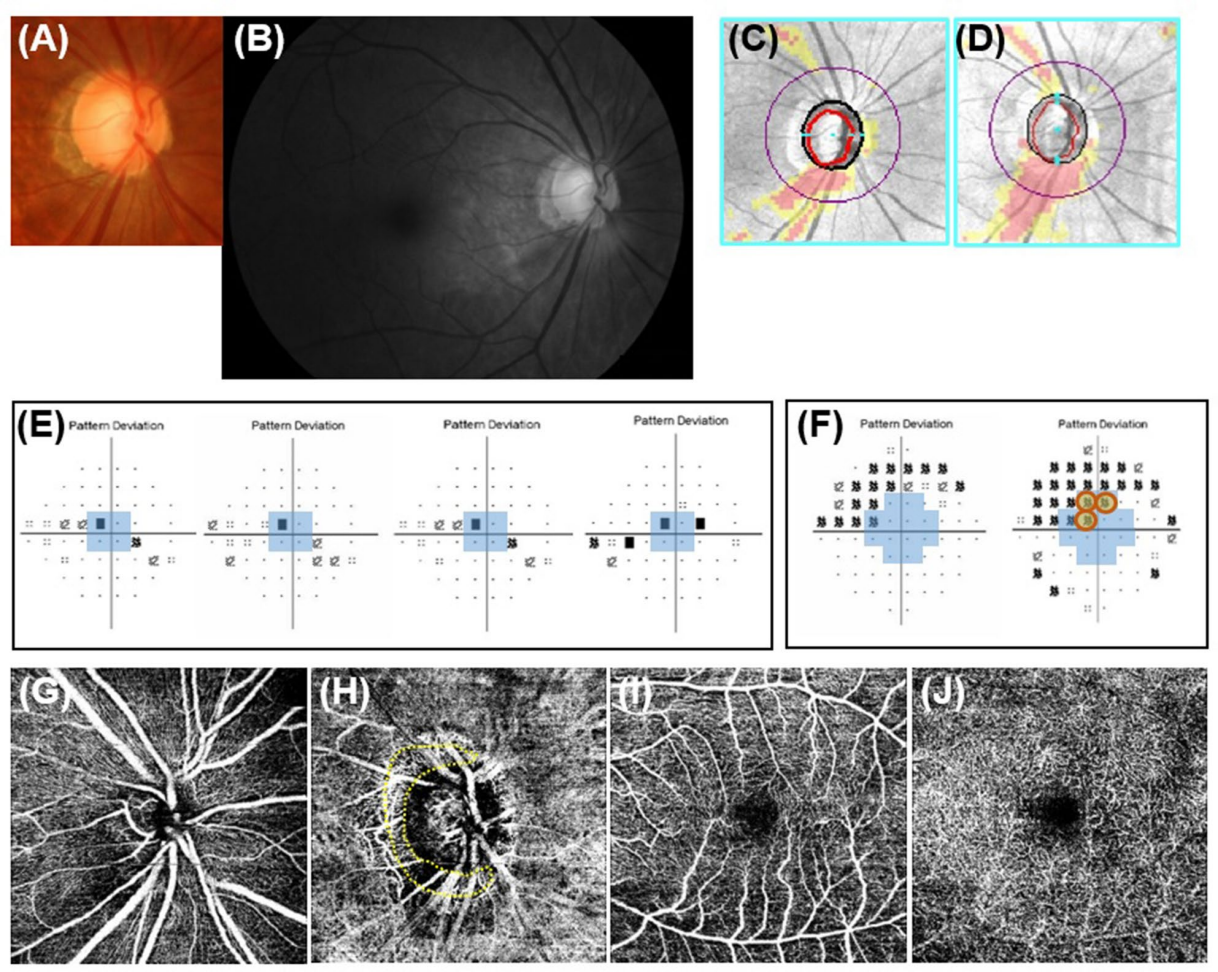
Representative case. A 69-year-old woman with myopia was diagnosed with normal-tension glaucoma. (**A**) and (**B**) Glaucomatous damage presenting as an inferior localized retinal nerve fiber layer defect and disc hemorrhage in the right eye. (**C**) and (**D**) The inferior localized retinal nerve fiber layer defect had widened at follow-up showing progression. (**E**) Serial images of 24-2 visual field (VF) showed initial parafoveal scotoma and VF progression was not apparent on 24-2 VF at follow-up. (**F**) However, innermost 10-2 VF progression was found on 10-2 VF. Optical coherence tomography angiography of the superficial layer around the optic disc (**G**), choroidal layer (**H**), superficial macular layer (**I**), and deep macular layer (**J**) showed decreased choroidal vessel density in the peripapillary atrophy region (yellow dotted area) in the right eye.

A representative case is shown in Fig. 5. A 69-year-old woman with myopia had NTG. Glaucomatous damage presented as an inferior localized RNFL defect and disc hemorrhage in the right eye. The visual fields showed initial paracenral scotoma on 24-2 VF and innermost VF progression was found on 10-2 VF. The choroidal VD in the PPA region was decreased in the right eye.

## Discussion

There have been reports that NTG appear to have deeper, more localized scotomas than POAG. However, the precise mechanism for this is not fully understood. Recent reports have shown that glaucoma patients with vascular risk factors may present with initial paracentral scotomas and progression in this region^11–13^. Several studies have reported disc hemorrhage, autonomic dysfunction, migraine, orthostatic hypotension, and Raynaud’s phenomenon as risk factors associated with central VF progression^14,15^. Here, we clearly showed that NTG is associated with initial paracentral scotomas closer to the region of fixation than POAG. In addition, NTG with disc hemorrhage had scotomas closer to the region of fixation, indicating that NTG with vascular risk factors may present with scotomas threatening vision. This can be explained by the contribution of vascular risk factors to disturbance of the blood flow to the optic disc, resulting in preferential damage to the central region where metabolic demand is high. Superior was more common than inferior initial paracentral scotoma, which we observed from the averaged total deviation map of the VFs of all patients included in the study. These findings were consistent with previous reports showing that superior hemi-field defect in the central region is more common in NTG patients with unstable ocular perfusion pressure.

Risk factors associated with central VF progression were diagnosis of NTG, presence of disc hemorrhage, and choroidal VD of the β-zone PPA region on OCT-A. The findings of this study suggested that vascular risk factors may contribute to progression near the region of fixation in NTG. Disc hemorrhage is a significant vascular risk factor for glaucoma development and progression^20–22^. The Ocular Hypertension Treatment Study found that DH was a risk factor for glaucoma development in ocular hypertensive eyes^22^. The Early Manifest Glaucoma Trial and Collaborative Normal-Tension Glaucoma Study showed that DH was significantly associated with glaucoma progression^20,23^. Our previous OCT-A study revealed VD defects in the choroidal perfusion where disc hemorrhage was found, which was related to glaucoma progression in this region. These observations suggest that compromised blood supply to the deep optic nerve head structures triggered vascular changes facilitating disc hemorrhage and further glaucoma progression.

Recent OCT-A studies reported that deep choroidal VD defects were frequently found in glaucomatous eyes, and were topographically related to glaucomatous damage^12–25^. Lee et al*.* reported that the presence of deep choroidal VD defect was a strong predictor of central VF damage in glaucoma patients^19^. The precise causal mechanism of deep choroidal VD defect in glaucoma remains unclear. However, it has been reported that systemic factors, such as cold extremities, migraine, and lower ocular perfusion pressure, were associated with the presence of deep choroidal VD defects, suggesting that systemic vascular factors may contribute to the development of these OCT-A findings. Especially, in terms of innermost 10-2 VF progression, which we defined as progression within the 12 points on the 10-2 VF, diagnosis of NTG and reduced choroidal VD of the β-zone PPA region on OCT-A were significantly associated with progression in this region. This study confirmed that deep choroidal VD is important in the central VF function of NTG patients, especially closer to the region of fixation. Reduced deep choroidal VD was a significant risk factor for progression of initial paracentral scotomas in both 24-2 and 10-2 VF tests. In NTG patients presenting with deep choroidal VD defects or reduced choroidal VD on OCT-A of the optic disc, we should consider that this vascular risk factor may contribute to VF progression threatening vision.

There have been reports that macular VD is related to central visual function in glaucoma. The foveal avascular zone have been shown to affect visual function in glaucoma using OCT-A^26,27^. Kwon et al*.* demonstrated that patients with a central visual defect had an enlarged foveal avascular zone and the area was significantly related to the severity of central scotoma^28^. In addition, Penteado et al*.* suggested superficial macular VD as a good functional parameter of central VF^29^. Our previous study showed that decreased deep macular VD was a risk factor for central scotoma in glaucoma patients. These findings support the suggestion that visualizing microvascular incompetence in the macular region may be important for predicting central function in glaucoma patients. The present study added that imaging the optic disc using OCT-A may also be important to monitor initial paracentral scotomas in glaucoma patients. Initial paracentral scotoma has been reported to be a factor related to future glaucoma progression^30,31^. The presence of central VF damage at baseline was significantly associated with more rapid global progression. These studies suggested that the pathogenesis for central scotomas in NTG may involve vascular dysregulation and ischemic-reperfusion damage^32^. Glaucoma patients with vascular risk factors as their pathogenesis may show more rapid and global progression. Therefore, glaucoma patients presenting with initial paracentral scotomas should be evaluated with regard to vascular risk factors, including OCT-A findings, and patients with related risk factors may have to be monitored more closely. Patients with a diagnosis of NTG, the presence of disc hemorrhage, thinner baseline GC/IPL thickness, and reduced choroidal VD in the β-zone PPA region should be considered to have risk of glaucoma progression even in the central VF region threatening vision.

This study had several limitations. First, OCT-A imaging itself still has certain limitations. Retinal vessel signals evident on en face, deep-layer OCT-A images make it difficult to measure precisely the choroidal VD. This should be considered when evaluating OCT-A images. However, recent studies have reported that measurement repeatability and reproducibility were good in intra- and intersession examinations^33–37^. We included patients with a minimum of five VFs, and these examinations were usually performed at 6–12-month intervals. More frequent testing is suggested using linear regression to calculate the rate of progression or detect VF progression. Considering these issues, we analyzed global mean deviation (MD) rates and applied a cut-off to define progression in both 24-2 and 10-2 VF tests. The cut-off value is not a definite definition for VF progression and this should therefore be taken into consideration when interpreting the results.

In conclusion, initial paracentral scotomas in NTG tended to progress closer to the region of fixation, which should be monitored closely. VF progression in the central VF region was related to the diagnosis of NTG, presence of disc hemorrhage, thinner baseline GC/IPL thickness, and reduced choroidal VD in the β-zone PPA region using OCT-A. We should evaluate these vascular risk factors in NTG patients presenting with initial paracentral scotoma to avoid vision threatening progression of glaucoma.

## Methods

Methodologies used in this study were conducted by referring our previous study^38^. In this prospective, observational study, glaucoma patients with initial paracentral scotoma underwent SAP 24-2 and SAP 10-2 VF tests (Carl Zeiss Meditec, Dublin, CA) at the glaucoma clinic of Seoul St. Mary’s Hospital, College of Medicine, Catholic University of Korea, Seoul, Republic of Korea, between November 2017 and August 2020. This study adhered to the Declaration of Helsinki and was approved by the Institutional Review Board of Seoul St. Mary’s Hospital. All patients provided informed consent.

As an initial evaluation, all patients performed a comprehensive ophthalmologic examination, slit-lamp biomicroscopy, Goldmann applanation tonometry, central corneal thickness measurement using ultrasound pachymetry (Tomey Corporation, Nagoya, Japan), gonioscopic examinations, axial length measurement using IOL Master (Carl Zeiss Meditec, Jena, Germany), stereoscopic optic disc photography, dilated stereoscopic examination of the ONH (optic nerve head), red-free photography (VX-10; Kowa Optimed, Tokyo, Japan), Cirrus SD-OCT scans (Carl Zeiss Meditec), and measurement of best-corrected visual acuity^38^.

All patients had to have newly diagnosed glaucoma without previous treatment. A diagnosis of normal-tension glaucoma (NTG) was made based on the following criteria: glaucomatous optic disc changes (such as diffuse or localized rim thinning, disc hemorrhage, notching or acquired pitting of the optic nerve, and a vertical cup-to-disc ratio higher than that of the other eye by > 0.2), or localized retinal nerve fiber layer (RNFL) defects on disc and red-free photography confirmed by two glaucoma specialists (EKK, HYP); an open angle on gonioscopic examination; best-corrected visual acuity better than 20/30; and maximum IOP < 22 mmHg (without glaucoma medications) during repeated measurements obtained on different days^11^. Diagnosis of primary open-angle glaucoma (POAG) was made based on the NTG criteria with the exception of IOP > 21 mmHg (without glaucoma medications), even just once during repeated measurements obtained on different days.

Patients were excluded on the basis of any of the following criteria: a history of systemic or neurological disease that may affect the VF; a history of ischemic optic nerve disease; a history of retinal disease including diabetic or hypertensive retinopathy; a history of eye trauma or surgery, with the exception of uncomplicated cataract surgery^11^. During follow-up period, there was no patient who had cataract surgery, and we excluded eyes presenting cataract progression. Cataract progression was determined using Lens Opacity Classification System (LOCS) III, and eyes showing nuclear color grade 4, nuclear opalescence grade 4, cortical cataract grade 3 or posterior sub-capsular cataract grade 2 were excluded^39^. If both eyes of a patient met the inclusion criteria and did not meet the exclusion criteria, one eye was randomly chosen for the study.

Two glaucoma specialists (EKK and HYP) evaluated the serial disc and red-free photographs during the total follow-up period in each patient. During follow-up, serial disc and red-free fundus photographs were taken at intervals of 6–12 months regularly^25^. Disc hemorrhage (DH) was defined as a splinter-like hemorrhage or isolated flame-shaped on the optic disc or peripapillary area extending to the border of the optic disc^25^.

### VF test and criteria for the presence of an initial paracentral scotoma

Assessment of visual function was conducted as in our previous study^7^. Patients underwent SAP 24-2 and 10-2 VF tests every 6 months for at least 24 months. In the 24-2 VF tests, three or more contiguous points (5% depressed from normative database) within the 10° region or one or more points (1% depressed from normative database) with no abnormality outside the central 10° region was defined as initial paracentral scotoma. In the 10-2 VF test, the presence of paracentral scotoma was defined as three or more contiguous points (5%, 5%, and 1% or 5%, 2%, and 2% depressed from normative database). This definition was modified from previous reports by Traynis et al*.*^4^ and Ehrlich et al*.*^20^ To analyze VF progression, the VF points depressed to < 5%, < 2%, < 1%, or < 0.5% on the 24-2 VF test, and the VF points depressed to < 5%, < 2%, or < 1% on the 10-2 VF test from the total deviation plot were evaluated. All 10-2 and 24-2 VF tests analyzed were required to have fixation losses, false-positives, and false-negatives ≤ 25%.

### Optical coherence tomography angiography measurements

OCT angiography measurements were conducted as in our previous study^17,18^. The ONH and parapapillary region were imaged using a commercial, swept-source OCT-A device (DRI OCT Triton, Topcon). The DRI OCT Triton system employed a swept source laser with a wavelength of 1050 nm and scan speed of 100,000 A-scans per second using the Topcon OCT angiography ratio analysis algorithm. To reduce motion artifacts during imaging, an active eye tracker was used. OCT-A of the DRI OCT generated en face images via automated layer segmentation around the optic nerve head into four layers: superficial, vitreoretinal, radial capillary network, and parapapillary choroidal layers. The whole images of the superficial layers and the deep layer parapapillary choroidal microvasculature in the β-zone peripapillary atrophy (PPA) region were evaluated using en face images generated by automated layer segmentation of signals from the retinal pigment epithelium that extended to the outer border of the sclera. The boundaries of the optic disc and β-zone PPA were delineated for measurement of choroidal VD in the β-zone PPA region. Eyes in which the optic disc or β-zone PPA could not be clearly delineated as well as eyes without β-zone PPA on the en face images were excluded.

The superficial vascular layer analyzes signals from 2.6 μm below the internal limiting membrane to 15.6 μm below the junction between inner plexiform and inner nuclear layers (IPL/INL), and the deep vascular layer analyzes signals from 15.6 μm below IPL/INL to 70.2 μm below IPL/INL. The whole images of the superficial and deep vascular plexus were used for macular VD. Only clear images with quality scores > 30 without blurring attributable to motion were analyzed. ImageJ software (National Institutes of Health, Bethesda, MD) was used to calculate VD. The measurement of vascular density was conducted as in our previous study^17^. A binary slab was created according to the ImageJ “mean threshold” algorithm. After assigning black pixels (background) and white pixels (vessel), VD was calculated as the ratio of vessel pixels and the total area in each image. Two independent observers (EKK and DYS) measured each VD independently and were blinded to the clinical data. The mean values of two measurements were used for further analysis.

### Cross-sectional analysis

For cross-sectional analysis to show the location and pattern of the paracentral scotoma, numerical values of the total 10-2 VF tests during the whole follow-up period were averaged for each VF test point using the total deviation map, generating one average map for each subgroup. A linear grayscale was generated and applied to all maps. To show the progression pattern of the initial paracentral scotoma, same maps were generated using the 10-2 VF tests at the initial and final visit to show the progression pattern of initial paracentral scotoma between subgroups (NTG compared to POAG).

### Longitudinal analysis

To evaluate the change in initial paracentral scotomas, longitudinal analysis was performed using total deviation maps of 24-2 and 10-2 VFs. For this analysis, only eyes with five or more reliable VFs were included. We performed linear regression analysis to obtain the slope of the change of MD of the VF. We defined progression on 24-2 and 10-2 VF when the magnitude of MD slope was worse than − 1.0 dB per year with *P* ≤ 0.05 based on previous study^40–43^. Initial paracentral scotomas were classified according to their progression pattern on 10-2 VF. Compared to the outermost 10-2 VF progression (Fig. 1A, B), three or more new contiguous points (5%, 5%, and 1% or 5%, 2%, and 2% depressed from normative database) at the central 12 points on 10-2 VF were considered as innermost 10-2 VF progression (Fig. 1C, D).

### Statistical analysis

Baseline characteristics were compared between groups using the independent *t* test or chi-square test. Interobserver reproducibility was calculated by means of intraclass correlation coefficient (ICC) for qualification of vessel density (VD) calculation from OCT-A images. Logistic regression analyses were performed to identify risk factors for VF progression. The dependent variable was presence of progression on 24-2 VF or 10-2 VF tests. The independent variables were age, sex, diagnosis of NTG, axial length, untreated baseline IOP, mean treated IOP, central corneal thickness, presence of disc hemorrhage, average RNFL thickness, average GC/IPL thickness, mean deviation (MD), pattern standard deviation (PSD), and OCT-A parameters. Variables that were significant with *P* < 0.2 on univariate analysis were included in multivariate analysis. In these analyses, *P* < 0.05 was taken to indicate statistical significance. Statistical analyses were performed using SPSS (ver. 18.0; SPSS Inc., Chicago, IL).
